# Psychometric evaluation of a pediatric functional constipation symptom diary using randomized phase 3 clinical trial data

**DOI:** 10.1186/s41687-025-00945-7

**Published:** 2025-11-20

**Authors:** Julia Vishnevetsky, Masakazu Ando, Yanqing Xu, Xiaolan Ye, Jessica L. Abel

**Affiliations:** 1 AbbVie, Inc., Florham Park, NJ USA; 2 Ironwood Pharmaceuticals, Boston, MA USA; 3 AbbVie, Inc., North Chicago, IL USA

**Keywords:** Pediatric functional constipation, Patient-reported outcomes, Psychometric evaluation

## Abstract

**Background:**

Patient-reported outcomes (PROs) are required to assess the efficacy of treatments for functional constipation (FC), which is one of the most common functional gastrointestinal disorders in children. A novel PRO, the Pediatric Functional Constipation Symptom Diary (PFCSD), was developed to assess 7 core symptoms of FC (stool frequency, stool consistency, incomplete evacuation, straining, abdominal pain, abdominal bloating, and fecal incontinence) in pediatric FC patients. Here, we describe the psychometric analyses of the PFCSD using data from a phase 3 clinical trial of linaclotide (LIN-MD-64; NCT04026113) in children with FC.

**Methodology:**

This psychometric evaluation assessed the measurement properties (reliability, validity, responsiveness) and meaningful within-patient change thresholds for symptom scores derived from the PFCSD. Since spontaneous bowel movement (SBM) frequency and stool consistency (measured using the pediatric Bristol Stool Form Scale in the PFCSD) were key endpoints for the phase 3 clinical trial of linaclotide, scores from these 2 items were the primary focus of these psychometric analyses. Daily and weekly responses on the PFCSD were assessed across a 14-week period from 328 pediatric patients aged 6 to 17 years who met the modified Rome III criteria for child/adolescent FC.

**Results:**

Test-retest reliability, construct validity, and responsiveness of SBM frequency and stool consistency scores on the PFCSD were supported by this psychometric evaluation. Using intraclass correlation coefficients (ICCs), PFCSD scores on items measuring SBM frequency (ICC = 0.91) and stool consistency (ICC = 0.56) were consistent across repeated administrations. Convergent and discriminant correlations underscored the validity of SBM frequency and stool consistency scores on the PFCSD. The meaningful change threshold for weekly SBM frequency was identified as a change from baseline of ≥ 2 SBMs. For stool consistency, the meaningful change threshold ranged between + 0.8 to + 1.7 on the pediatric Bristol Stool Form Scale.

**Conclusions:**

The PFCSD is a novel, fit-for-purpose PRO measure evaluating key symptoms experienced by patients with FC aged 6 to 17 years. These analyses support the reliability, validity, and responsiveness of SBM frequency rate and stool consistency scores on the PFCSD in this target population and provide further guidance for interpreting meaningful changes in these scores at the individual level.

**Supplementary Information:**

The online version contains supplementary material available at 10.1186/s41687-025-00945-7.

## Background

Functional constipation (FC) is a chronic, idiopathic condition characterized by infrequent bowel movements (BMs) associated with painful defecation and straining due to hard and/or large stools [1]. FC affects approximately 12.1% of children in the United States, making it one of the most common functional gastrointestinal disorders in children [2]. Key symptoms of FC include BM symptoms such as infrequent BMs, hard stools, incomplete evacuation, and straining; and abdominal symptoms such as pain and bloating [3, 4]. Impacts of FC symptoms, including behavioral and emotional challenges, can significantly impair a child’s quality of life [5, 6].

Since there are no established clinical or biological measures to evaluate FC, measuring treatment effects on the symptoms patients experience remains the primary method of assessing efficacy of FC treatments and must be assessed exclusively through patient-reported outcomes (PROs). The Pediatric Functional Constipation Symptom Diary (PFCSD), a novel, fit-for-purpose, patient-reported electronic diary (eDiary), assesses 7 core symptoms of FC: stool frequency, stool consistency, incomplete evacuation, straining, abdominal pain, abdominal bloating, and fecal incontinence [3, 4]. The development of the PFCSD included extensive qualitative research with children with FC and their caregivers [3, 4] as well as input from the US Food and Drug Administration’s (FDA’s) Divisions of Gastroenterology and Clinical Outcome Assessment to create an accessible tool that facilitates accurate self-reporting of FC symptoms to evaluate treatment outcomes.

The PFCSD was implemented in a phase 3 randomized controlled trial of linaclotide in pediatric patients with FC aged 6 to 17 years (LIN-MD-64; NCT04026113) to support primary and secondary endpoints assessing the efficacy of linaclotide on improvements in spontaneous BM (SBM) frequency and stool consistency [7]. Positive results from this trial, along with data supporting the use of the PFCSD in this target patient population, supported the approval of linaclotide as the first FDA-approved pharmaceutical treatment for pediatric patients with FC aged 6 to 17 years [8]. To confirm PFCSD measurement properties and support the use of PFCSD scores as endpoints in future trials of pediatric FC, a comprehensive psychometric evaluation of the PFCSD was conducted using data from the LIN-MD-64 trial. Here, we describe the confirmatory psychometric analyses measuring the reliability, validity, responsiveness, and individual change thresholds of the BM and abdominal symptom scores derived from the PFCSD.

## Methods

### Study design

This psychometric evaluation used blinded data from the LIN-MD-64 trial (NCT04026113), which assessed the efficacy and safety of linaclotide in 328 pediatric participants in the modified intention-to-treat (mITT) population (i.e., all participants who were randomized and received at least 1 dose of the double-blind study intervention) aged 6 to 17 years who fulfilled modified Rome III criteria for child/adolescent FC. Additional eligibility criteria are described in Di Lorenzo et al. [7]. Trial randomization targeted a 1:1 ratio of participants receiving either linaclotide or placebo and was stratified by age group only (6 to 11 years of age and 12 to 17 years of age), with a minimum of 40% of participants in each age group. Trial participants and researchers remained blinded to treatment assignment for the duration of the trial (i.e., double-blinded).

The trial was organized into 4 periods: Screening, Pre-intervention, Intervention, and Post-intervention. These psychometric analyses used eDiary responses from the Pre-intervention and Intervention periods. Treatment assignment was excluded from all analyses. During the Pre-intervention period, which lasted 2 to 3 weeks, participants completed the PFCSD twice daily and the Patient Global Impression of Severity (PGIS) measure weekly. The 2 weeks of the Pre-intervention period preceding randomization defined the Baseline period (Week − 2 and Week − 1) for twice-daily PFCSD scores and weekly PGIS scores. The Intervention period began with randomization, at which time participants started completing the weekly Patient Global Impression of Change (PGIC) measure. Throughout the 12-week Intervention period (Week 1 to Week 12), participants completed the PFCSD twice daily and the PGIS and PGIC measures weekly.

### Measures

The daily PFCSD items measured BM characteristics, fecal incontinence, abdominal symptoms, and rescue medication use in a morning (i.e., “from bedtime last night until now”) and evening (i.e., “from when you got up this morning until now”) administration. The PFCSD was available in 2 administration modes (self-completed and caregiver-administered), both of which contained identical items. The self-completed version was available for all participants (ages 6–17). If the participant was 6 to 11 years of age and had difficulty reading or navigating the PFCSD without assistance, then the participant’s caregiver would read the questions and response choices verbatim from the PFCSD and enter the answer chosen by the participant (caregiver-administered version). Regardless of the chosen mode of administration, the PFCSD was considered a PRO measure because the participant reported on their health condition without change or interpretation by anyone else.

The PGIS and PGIC measures served as global anchors for the anchor-based psychometric evaluation of the PFCSD. The PGIS and PGIC were administered weekly, and each measure consisted of 2 items (“pooping problems” and “tummy problems”) with 5-point response scales. For the PGIS items, the scale ranged from “I have not had pooping/tummy problems” to “very bad.” For the PGIC items, the scale ranged from “a lot better” to “a lot worse.” PGIS items assessed participant symptom severity over the past 7 days of each week (14 weeks total) starting at Week − 2 (Baseline) and concluding at the end of Week 12 (final week of Intervention). PGIC items assessed change in participant symptoms over the past 7 days of each week (12 weeks total) starting at Week 1 (Intervention) and concluding at the end of Week 12. These weekly measures were completed by all participants aged 6 to 17 years (self-completed). For participants aged 6 to 11 years, another set of global anchors (administered weekly) was completed by the participant’s caregiver according to the caregiver’s observations of the child’s health condition (caregiver-observed). These caregiver-observed measures each consisted of 1 item (constipation symptoms for the global change anchor and constipation severity for the global severity anchor). The recall periods of the caregiver-observed items matched those of the respective weekly participant-completed diary items (e.g., the recall period for the caregiver-observed global severity item matched the PGIS recall period).

The primary efficacy endpoint of the LIN-MD-64 trial was the change from Baseline in the 12-week SBM frequency rate during Intervention. An SBM was defined as a BM that occurred in the absence of rescue medication (e.g., laxative, suppository, or enema) on the day before or day of the BM. Since the diary captured daily rescue medication use and BM occurrence, these data were combined to capture SBM frequency. The complete SBM (CSBM) frequency rate, defined as an SBM associated with a sense of complete evacuation, was also captured. The secondary endpoint was change from Baseline in 12-week stool consistency score measured by the pediatric Bristol Stool Form Scale included as part of the PFCSD, with responses ranging from type 1 (small hard lumps or balls) to type 7 (watery, looks like a milkshake). Because the change in SBM frequency and stool consistency scores derived from the PFCSD were the key efficacy endpoints of the LIN-MD-64 trial, responses to these PFCSD items were the focus of this confirmatory psychometric evaluation.

### Statistical analyses

All data were pooled, regardless of treatment group, and analyses were performed using SAS version 9.4 or higher (SAS Institute Inc.). In contrast to the efficacy analyses, the psychometric analyses relied on evaluating weekly scores instead of overall change from baseline. To calculate SBM frequency rate, the total number of events (i.e., SBMs) during a specific period was divided by the number of diary days for that period and multiplied by 7. Participants’ stool consistency score was computed as the mean of the non-missing, SBM-associated pediatric Bristol Stool Form Scale scores (morning and evening) from the eDiary during the period of interest. As such, derivations of the SBM frequency rate and stool consistency scores in this psychometric analysis (i.e., weekly scores) differed from the primary and secondary endpoints derived over the 12-week period of the LIN-MD-64 trial. For test-retest reliability, construct validity using known-groups methods, responsiveness, and score interpretation, weekly PFCSD scores were derived in the context of the global anchors. To ensure comparable recall periods for these anchor-based analyses, PFCSD scores were calculated over the 1-week period prior to global item completion. For the remaining psychometric analyses (i.e., construct validity correlations and sample descriptive characteristics of Baseline and Intervention), weekly PFCSD scores were based on the randomization date. Notably, while the sample size for the psychometric analyses that did not rely on anchors was the same as that for the efficacy analyses based on the mITT population (*n* = 328), the majority of the psychometric analyses were anchor-based. The sample size with nonmissing data for anchor-based analyses was smaller because both the PFCSD scores and anchor variables were required to compute weekly scores, and missing data were driven by missing responses to anchor variables.

Demographic and clinical variables were reported for each participant. Descriptive statistics were reported for each of the PFCSD items during the Baseline and Intervention periods. Statistical testing (2-sided independent samples t-test, α = 0.05) was used where appropriate. PFCSD scores computed as an average value over time (i.e., 12 weeks) were based on nonmissing responses. Inconclusive responses in the PFCSD (e.g., “I don’t know”) were treated as missing values. The PFCSD did not allow for incomplete data within an assessment, and if participants completely missed an assessment (i.e., did not record any response in the eDiary), missing responses were not imputed.

### Test-retest reliability

The test-retest reliability of the PFCSD was evaluated using intraclass correlation coefficients (ICCs), which were calculated by a 2-way mixed-effects regression model based on absolute agreement [9] using weekly average PFCSD scores from Week − 2 (test) and Week − 1 (retest) and from Week 11 (test) and Week 12 (retest). Only scores from participants who were considered stable—defined as those during the test-retest periods with identical scores to the PGIS anchor (i.e., “tummy problems” item for abdominal pain and bloating scores and “pooping problems” item for the remaining PFCSD scores)—were used for these analyses. ICCs ≥ 0.7, between 0.4 and 0.7, and < 0.4 indicate good test-retest reliability, moderate test-retest reliability, and poor test-retest reliability, respectively [10].

### Construct validity

Correlations between PFCSD item scores were used in an exploratory manner to evaluate convergent and discriminant validity. Inter-item correlations were calculated using the overall average of PFCSD item scores during Baseline (Week − 2 through Week − 1) and Intervention (Week 1 through Week 12). Due to the common symptom type, it was predicted that correlations among BM-related symptom scores would be moderate in size (0.3 ≤ |*r*|) based on Cohen’s criteria [11]. In contrast, it was hypothesized that correlations between BM-related symptom scores and abdominal symptom scores would be smaller in size (0.3 >|*r*|) since these pairs of scores measured different symptom types.

The construct validity of PFCSD scores was further evaluated using the known-groups method to test their ability to distinguish between high- and low-severity groups as defined by the PGIS anchor for “pooping problems” and “tummy problems” and by the caregiver-observed constipation severity anchor. For the PGIS anchors, high severity was defined as a response of “bad” or “very bad” and low severity as a response of “no problems” or “a little bad.” For the caregiver-observed constipation severity anchor, high and low severity groups were defined as a response of “severe” or “very severe” and “none” or “mild,” respectively. These analyses were conducted separately for PFCSD scores at Week − 1 and Week 12. A 2-sided, independent samples t-test was used to calculate p-values [11].

### Responsiveness

The ability of PFCSD scores to detect changes over time was evaluated by comparing participants defined as “improved” with those defined as “stable” and “worsened” based on the PGIS, PGIC, and caregiver-observed anchors. For instance, with the PGIS scores as an anchor, participants were classified as follows: improved = 1-category improvement or greater (i.e., “bad” to “a little bad”); stable = identical scores; and worsened = 1-category worsening or greater (i.e., “bad” to “very bad”). Based on the caregiver-observed constipation global severity anchor, classification of participants was as follows: improved = 1-category improvement or greater (i.e., “mild” to “none”); stable = identical scores; and worsened = 1-category worsening or greater (i.e., “mild” to “moderate”). Analysis of variance methods were used to model the change in PFCSD scores for each of the groups. Guyatt’s responsiveness statistic (GRS) was reported as an effect size comparing the improved group with the stable group and with the worsened group. The GRS is interpreted as small (0.2 to < 0.5), medium (0.5 to < 0.8), and large (≥ 0.8) [11, 12]. For groups defined using the PGIS or caregiver-observed constipation global severity anchors, Week − 1 was used as the first timepoint (baseline) and Week 12 was used as the second timepoint (intervention). For groups defined using the PGIC or caregiver-observed constipation global change anchors, changes in PFCSD scores from Week 11 to Week 12 were evaluated using the corresponding anchor at Week 12.

### Meaningful within-patient change thresholds

The meaningful change thresholds for SBM frequency rate and stool consistency PFCSD scores were estimated using anchor- and distribution-based methods. For anchor-based methods, the PGIS “pooping problems” item and the caregiver-observed severity item served as anchors, and a 1-category improvement was considered a meaningful change. Empirical cumulative distribution function (eCDF) and probability density function (PDF) plots were generated to show the change in SBM frequency rate and stool consistency scores across the “pooping problems” PGIS item (-3 to + 3 category improvement) and the caregiver-observed constipation global severity item from Week − 1 to Week 12 (-4 to + 4 category improvement). Additionally, the sensitivity, specificity, positive predictive value (PPV), and negative predictive value (NPV) were examined along the range of the PFCSD change score scale in classifying responders based on the anchors. Two distribution-based methods were applied to evaluate the meaningful change thresholds suggested by the anchor-based methods: the one-half standard deviation method and the standard error of measurement [13, 14].

## Results

### Participant demographics and characteristics

The patient population consisted of 328 children and adolescents, with a mean (standard deviation) age of 11.09 (3.14) years; most patients were female (55.2%), and 45.1% were of Hispanic or Latino ethnicity (Table 1). BM characteristics and abdominal symptoms associated with FC were recorded during the Baseline period and throughout the 12-week Intervention period using the PFCSD (Table 2).

**Table 1:** Participant characteristics

Characteristic	Frequency (%) (*N* = 328)
Age	
6–11	181 (55.18)
12–17	147 (44.82)
Sex	
Female	181 (55.18)
Male	147 (44.82)
Race	
American Indian or Alaska Native	1 (0.30)
Asian	5 (1.52)
Black or African American	86 (26.22)
Multiple	3 (0.91)
Native Hawaiian or other Pacific Islander	4 (1.22)
White	229 (69.82)
Ethnicity	
Not Hispanic or Latino	180 (54.88)
Hispanic or Latino	148 (45.12)
Administration mode	
Self-completed	219 (66.77)
Caregiver-administered	109 (33.23)

**Table 2:** PFCSD scores from week − 2 to week − 1 and week 1 to week 12

PFCSD score	Time	*N*	Mean (SD)
SBM frequency rate^a^	Week − 2 to Week − 1	328	1.22 (0.85)
	Week 1 to Week 12	328	2.85 (2.47)
Stool consistency	Week − 2 to Week − 1	276	2.37 (0.93)
	Week 1 to Week 12	318	3.37 (1.02)
CSBM frequency rate^b^	Week − 2 to Week − 1	328	0.57 (0.74)
	Week 1 to Week 12	328	1.95 (2.12)
Fecal incontinence	Week − 2 to Week − 1	328	0.07 (0.15)
	Week 1 to Week 12	327	0.07 (0.15)
Straining	Week − 2 to Week − 1	280	2.50 (1.07)
	Week 1 to Week 12	320	1.53 (1.00)
Abdominal pain	Week − 2 to Week − 1	328	1.10 (1.07)
	Week 1 to Week 12	328	0.72 (0.95)
Abdominal bloating	Week − 2 to Week − 1	328	1.15 (1.11)
	Week 1 to Week 12	328	0.71 (1.00)

BM = bowel movement; CSBM = complete SBM; PFCSD = Pediatric Functional Constipation Symptom Diary; SBM = spontaneous BM; SD = standard deviation

^a^ Defined as a BM that occurred in the absence of rescue medication such as a laxative, enema, or suppository on the day before or day of the BM

^b^ Defined as SBMs that were associated with a sense of complete evacuation

Notes: Week − 2 to Week − 1 defines the Baseline period, and Week 1 to Week 12 defines the Intervention period. The PFCSD captured evacuation completeness, which was combined with BM occurrence and rescue medication data to capture CSBMs

### Test-retest reliability

The ICC for SBM frequency rate exceeded the 0.70 threshold for good test-retest reliability (ICC = 0.91), and the ICC for stool consistency exceeded the minimum threshold for moderate test-retest reliability (ICC = 0.56) for weeks 11 to 12 (Table 3). In contrast, the test-retest reliability was poor (ICC < 0.4) from Week − 2 to Week − 1 for both SBM frequency rate (ICC = 0.05) and stool consistency (ICC = 0.35). This low ICC value for SBMs was likely influenced by the study inclusion criteria that limited the range of SBM values prior to randomization (i.e., fewer than 3 SBMs per week). Similarly, the study inclusion criteria reducing the variability of stool consistency scores (i.e., no more than 1 stool of type 6 or 7 permitted prior to randomization) likely contributed to the lower ICC value for stool consistency. The remaining BM symptoms and all abdominal symptoms exceeded the good test-retest reliability threshold for weeks 11 to 12 (Table 3). For these symptoms, only the CSBM frequency rate (ICC = 0.35) and straining (ICC = 0.64) did not meet the 0.70 threshold for good test-retest reliability for weeks − 2 to -1 (Table 3).

**Table 3:** Test-retest reliability for PFCSD scores

PFCSD item	Time	*N*	ICC (95% CI)
SBM frequency rate	Week − 2 to Week − 1	114	0.05 (-0.14 to 0.23)
	Week 11 to Week 12	57	0.91 (0.85 to 0.95)
Stool consistency	Week − 2 to Week − 1	58	0.35 (0.10 to 0.55)
	Week 11 to Week 12	45	0.56 (0.32 to 0.73)
CSBM frequency rate	Week − 2 to Week − 1	114	0.35 (0.17 to 0.50)
	Week 11 to Week 12	57	0.93 (0.89 to 0.96)
Fecal incontinence	Week − 2 to Week − 1	114	0.70 (0.60 to 0.79)
	Week 11 to week 12	57	0.72 (0.57 to 0.83)
Straining	Week − 2 to Week − 1	64	0.64 (0.46 to 0.76)
	Week 11 to Week 12	45	0.89 (0.82 to 0.94)
Abdominal pain	Week − 2 to Week − 1	102	0.89 (0.85 to 0.93)
	Week 11 to Week 12	62	0.96 (0.93 to 0.98)
Abdominal bloating	Week − 2 to Week − 1	102	0.89 (0.84 to 0.93)
	Week 11 to Week 12	62	0.98 (0.96 to 0.99)

CI = confidence interval; CSBM = complete SBM; ICC = intraclass correlation coefficient; PFCSD = Pediatric Functional Constipation Symptom Diary; SBM = spontaneous bowel movement

### Construct validity

#### Convergent and discriminant validity

The construct validity of PFCSD scores was evaluated through inter-item correlations from Week − 2 through Week − 1 (Baseline period) and Week 1 through Week 12 (Intervention period) (Table 4). SBMs were strongly correlated with CSBMs (Baseline *r* = 0.65; Intervention *r* = 0.82) and moderately correlated with straining during Intervention (*r* = -0.32) but not during Baseline (*r* = -0.22). Stool consistency was moderately correlated with straining (Baseline *r* = -0.44; Intervention *r* = -0.49) but had less correlation with SBMs (Baseline *r* = 0.09; Intervention *r* = 0.22) and CSBMs (Baseline *r* = 0.15; Intervention *r* = 0.21). The lower correlations observed during Baseline may reflect the reduced variability in BM-related scores prior to randomization due to the study inclusion criteria.

**Table 4:** Inter-item correlations for PFCSD scores from weeks − 2 to -1 and weeks 1 to 12

PFCSD item	SBM frequency rate	Stool consistency	CSBM frequency rate	Fecal incontinence	Straining	Abdominal pain	Abdominal bloating
SBM frequency rate	-	0.09	0.65	-0.05	-0.22	-0.08	-0.13
Stool consistency	0.22	-	0.15	-0.05	-0.44	-0.28	-0.33
CSBM frequency rate	0.82	0.21	-	-0.04	-0.40	-0.31	-0.36
Fecal incontinence	0.06	0.10	0.05	-	-0.00	0.10	0.10
Straining	-0.32	-0.49	-0.44	-0.04	-	0.42	0.47
Abdominal pain	-0.15	-0.12	-0.31	0.03	0.47	-	0.78
Abdominal bloating	-0.15	-0.21	-0.32	0.02	0.48	0.82	-

CSBM = complete SBM; PFCSD = Pediatric Functional Constipation Symptom Diary; SBM = spontaneous bowel movement

Notes: Correlations for Week − 2 through Week − 1 (Baseline) are above the diagonal, and correlations for Week 1 through Week 12 (Intervention) are below the diagonal. All correlations are Pearson. 276 *≥* N *≥* 328

During the Intervention period, both SBMs and stool consistency mostly exhibited stronger correlations with other BM-related symptoms than with abdominal symptoms; fecal incontinence, which was poorly correlated with every symptom type, was the exception. Abdominal pain and bloating symptoms exhibited much stronger correlations between each other than with BM-related symptoms during both Baseline and Intervention periods (Table 4). Overall, the inter-item correlation patterns were consistent with our hypothesis and support the construct validity of the PFCSD scores for the key endpoints of SBM frequency rate and stool consistency.

#### Known-groups method

Significant differences were found between high- and low-severity groups, as defined using the PGIS “pooping problems” item, for both SBM frequency rate and stool consistency at Week 12 (*P* < 0.001), and the corresponding effect sizes were medium (Table 5). Similarly, significant differences were found between high- and low-severity groups for CSBM frequency rate and stool consistency at Week 12 (*P* < 0.001), with medium and large effect sizes, respectively. At Week − 1, small and statistically nonsignificant differences were identified for SBM frequency rate and stool consistency, whereas significant differences were found for CSBM frequency rate (*P* = 0.009) and straining (*P* < 0.001). The known-groups differences were smaller during Week − 1, at which time the variability of scores was reduced due to study eligibility criteria. All abdominal symptoms exhibited significant differences between high- and low-severity groups, as defined using the PGIS “tummy problems” item, at Week − 1 and Week 12 with large effect sizes (Table 5). When using the caregiver-observed global severity anchor, the small sample sizes for the low-severity group at Week − 1 and the high-severity group at Week 12 made it difficult to interpret P values. The effect size for SBM frequency rate was small at Week − 1 and large at Week 12, while the effect sizes for stool consistency were small at both time points (Table S-1). Taken together, these results further support the construct validity of SBM frequency rate and stool consistency scores.

**Table 5:** Construct validity using known-groups method with the PGIS anchors

PFCSD item	Time	Groups	*N*	Mean	SD	Effect size	*P* value
SBM frequency rate	Week − 1	High severity	143	1.41	1.08	0.00	0.894
		Low severity	82	1.43	1.25		
	Week 12	High severity	28	1.50	1.75	0.09	< 0.001
		Low severity	89	3.61	3.12		
Stool consistency	Week − 1	High severity	111	2.31	1.18	0.02	0.073
		Low severity	57	2.64	1.01		
	Week 12	High severity	17	2.65	1.42	0.12	< 0.001
		Low severity	77	3.78	1.13		
CSBM frequency rate	Week − 1	High severity	143	0.57	0.92	0.03	0.009
		Low severity	82	0.93	1.09		
	Week 12	High severity	28	0.93	1.41	0.09	< 0.001
		Low severity	89	2.85	2.86		
Fecal incontinence	Week − 1	High severity	143	0.08	0.17	0.00	0.305
		Low severity	82	0.06	0.14		
	Week 12	High severity	28	0.07	0.12	0.00	0.931
		Low severity	89	0.07	0.19		
Straining	Week − 1	High severity	113	2.81	1.03	0.21	< 0.001
		Low severity	60	1.68	1.06		
	Week 12	High severity	17	2.70	1.15	0.20	< 0.001
		Low severity	78	1.27	1.10		
Abdominal pain	Week − 1	High severity	102	1.62	1.21	0.24	< 0.001
		Low severity	123	0.55	0.65		
	Week 12	High severity	23	1.72	1.22	0.38	< 0.001
		Low severity	94	0.33	0.54		
Abdominal bloating	Week − 1	High severity	102	1.74	1.22	0.25	< 0.001
		Low severity	123	0.59	0.77		
	Week 12	High severity	23	1.64	1.31	0.32	< 0.001
		Low severity	94	0.32	0.57		

PFCSD = Pediatric Functional Constipation Symptom Diary; PGIS = Patient Global Impression of Severity; SD = standard deviation

Notes: This analysis used PGIS anchor items “pooping problems” and “tummy problems.” PGIS items were selected to correspond to the PFCSD item as follows: “tummy problems” for abdominal pain and abdominal bloating PFCSD scores, and “pooping problems” for the remaining PFCSD scores. Severity groups were defined by these anchor items for Week − 1 and Week 12. High severity is defined as a response of “bad” or “very bad.” Low severity is defined as a response of “no problems” or “a little bad.”

### Responsiveness

Responsiveness methods were applied to confirm that participants who experienced either global improvement, no change, or worsening of symptoms also reported this on the twice-daily PFCSD. The effect sizes, as calculated by GRS, for comparing the change groups within each PFCSD item are presented in Table 6. The GRS was small for SBM frequency rate, medium for CSBM frequency rate, and large for stool consistency when comparing Improved versus Stable groups and Improved versus Worsened groups based on PGIS items. When using PGIC items to define change groups, the GRS was small only for stool consistency and straining for the Improved versus Worsened groups, but the sample size in each Worsened group made the GRS difficult to interpret (Table S-2). The GRS for SBM frequency rate was negative, reflecting a relationship in the opposite direction with the change anchor (Table S-2). For caregiver-observed global severity, the GRS for Improved versus Stable groups was small for SBM frequency rate and large for stool consistency (Table S-3). The small sample sizes of the Worsened groups made the comparison between the Improved and Worsened groups difficult to interpret. For caregiver-observed global change, the GRS for SBM frequency rate for Improved versus Stable groups was negative, reflecting a relationship in the wrong direction with the anchor (Table S-4). The sample size of the Stable group for stool consistency was too small to interpret the Improved versus Stable GRS, and the Worsened group sample sizes were too small (*n* = 1) to compute the GRS for Improved versus Worsened groups for all PFCSD items. Particularly for the global severity anchors, these results indicate that stool consistency scores can detect changes over time and demonstrate that scores for SBM frequency rate can also reflect changes over time, although with less power.

**Table 6:** Responsiveness statistics for change in PFCSD scores from weeks − 1 to 12 using PGIS anchors

PFCSD item	Groups	*N*	Mean	SD	GRS improved vs. stable	GRS improved vs. worsened
SBM frequency rate	Improved	53	1.94	2.42	0.20	0.32
	Stable	29	1.21	3.77		
	Worsened	12	1.00	2.95		
Stool consistency	Improved	35	1.20	1.41	1.12	1.70
	Stable	13	0.19	0.90		
	Worsened	4	0.19	0.59		
CSBM frequency rate	Improved	53	2.15	2.05	0.45	0.46
	Stable	29	0.76	3.07		
	Worsened	12	1.00	2.52		
Fecal incontinence	Improved	53	-0.01	0.16	0.19	-0.36
	Stable	29	-0.04	0.19		
	Worsened	12	0.06	0.18		
Straining	Improved	36	-1.34	1.29	-1.52	-1.92
	Stable	14	0.33	1.10		
	Worsened	4	0.33	0.87		
Abdominal pain	Improved	44	-0.61	1.10	-0.68	-0.93
	Stable	35	-0.17	0.64		
	Worsened	15	0.09	0.75		
Abdominal bloating	Improved	44	-0.77	0.91	-0.70	-0.93
	Stable	35	-0.28	0.70		
	Worsened	15	-0.08	0.75		

CSBM = complete SBM; GRS = Guyatt’s responsiveness statistic; PFCSD = Pediatric Functional Constipation Symptom Diary; PGIS = Patient Global Impression of Severity; SBM = spontaneous bowel movement; SD = standard deviation

Notes: This analysis used PGIS anchor items “pooping problems” and “tummy problems.” PGIS items were selected to correspond to the PFCSD item as follows: “tummy problems” for abdominal pain and abdominal bloating PFCSD scores, and “pooping problems” for the remaining PFCSD scores. Change is computed as the later timepoint (improved) minus the earlier timepoint (stable or worsened). Improved = one category improvement or greater. Stable = no change. Worsened = one category worsened or greater

### Meaningful within-patient change

**Fig. 1:**
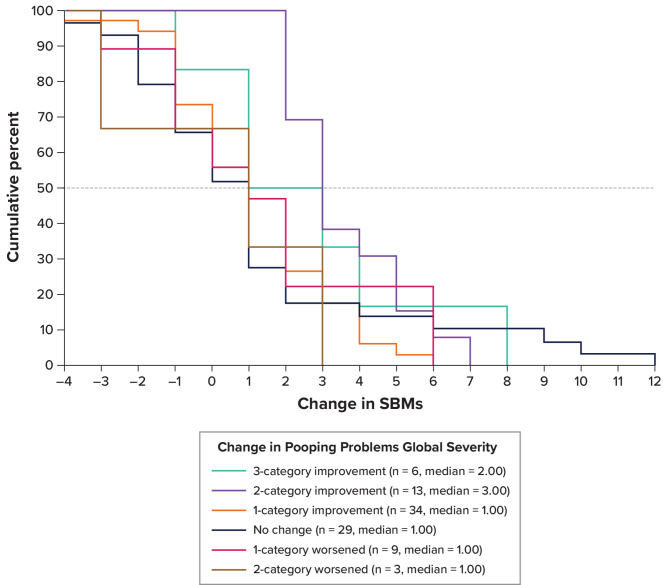
eCDF of Change in PFCSD SBMs and PGIS Pooping Problems: Weeks − 1 to 12. eCDF = empirical cumulative distribution function; PFCSD = Pediatric Functional Constipation Symptom Diary; PGIS = Patient Global Impression of Severity; SBM = spontaneous bowel movement. Notes: Change was computed as the later time point minus the earlier time point. Positive change indicates improvement. Pooping problems global severity was participant completed and available for all participants

PFCSD items for SBM frequency rate and stool consistency were used to define primary and secondary endpoints in the LIN-MD-64 trial (NCT04026113). Thus, a key focus of this psychometric evaluation was to define meaningful within-patient change thresholds for these 2 PFCSD item scores. For SBM frequency rate, the eCDF for the PGIS item “pooping problems” showed a median change of 1.0 SBM for 1-category improvement, which overlapped with the no-change group at the 50% line, suggesting that a threshold of 1.0 SBM may be too low to delineate true change (Fig. 1). The 2-category improvement group had a median of 3.0 SBMs, which was larger than that for 1-category improvement. The corresponding PDF showed that the probability of being in the 1-category improvement group reached its peak around a value of 0.7 SBMs and exceeded that of adjacent groups around a value of 1.0 SBM (Figure S-1). The classification statistics predicting 1-category improvement showed that sensitivity and specificity were simultaneously maximized at a threshold of 2.0 SBMs (60% and 70%, respectively), while PPV and NPV were also simultaneously maximized at a threshold of 2.0 SBMs (73% and 58%, respectively) (Figure S-2). For caregiver-observed global severity of constipation, the eCDF showed a median for 1-category improvement of 2.0 SBMs, which did not overlap with the no-change group (median = 0.50) (Figure S-3). The PDF showed that the probability of being in the 1-category improvement group peaked at 1.8 and exceeded that of adjacent groups around 1.5 (Figure S-4). The classification statistics predicting 1-category improvement showed that sensitivity and specificity were maximized at a threshold of 2 (62% and 74%, respectively), while PPV and NPV were also simultaneously maximized at a threshold of 2 (83% and 48%, respectively) (Figure FS-5). Thus, these analyses suggest a change from Baseline of ≥ 2 SBMs as a meaningful within-patient change threshold. The distribution-based analyses for SBM frequency rate are presented in Table S-5.

**Fig. 2:**
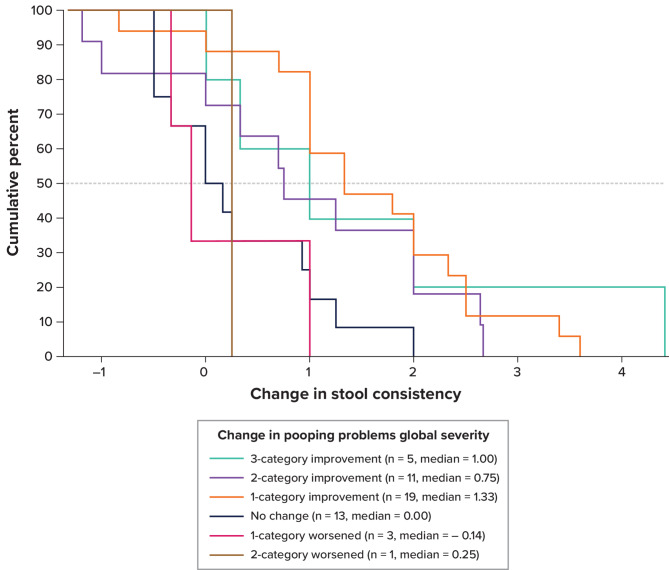
eCDF of Change in PFCSD Stool Consistency and PGIS Pooping Problems: Weeks − 1 to 12 eCDF = empirical cumulative distribution function; PFCSD = Pediatric Functional Constipation Symptom Diary; PGIS = Patient Global Impression of Severity. Notes: Stool consistency score was measured by the pediatric Bristol Stool Form Scale, which was included as part of the PFCSD. Change was computed as the later time point minus the earlier time point. Positive change indicates improvement. Pooping problems global severity was participant completed and available for all participants

For stool consistency, the eCDF for the “pooping problems” PGIS item showed a median for 1-category improvement of 1.33 on the pediatric Bristol Stool Form Scale, which did not overlap with the no-change group (median = 0.00) (Fig. 2). The 2-category improvement group had a median improvement of 0.75 on the pediatric Bristol Stool Form Scale, which was smaller than that for 1-category improvement and possibly an artifact of the 2-category improvement group’s small sample size (*n* = 11). The corresponding PDF (Figure S-6) showed that the 1-category improvement group reached its peak at 1.4, and its probability exceeded that of adjacent groups above values of 1.0. The classification statistics predicting 1-category improvement showed that sensitivity and specificity were simultaneously maximized at a threshold of 0.3 (77% and 71%, respectively), while PPV and NPV were also simultaneously maximized at a threshold of 0.3 (84% and 60%, respectively) (Figure S-7). For caregiver-observed global severity of constipation, the eCDF showed a median for 1-category improvement of 1.17, which did not overlap with the no-change group (median = 0.00) (Figure S-8). The PDF (Figure S-9) showed the 1-category improvement group at 1.3, and its probability exceeded that of adjacent groups between 0.8 and 1.7. The classification statistics predicting 1-category improvement showed sensitivity and specificity were maximized at a threshold of 0.3 (86% and 89%, respectively), while PPV and NPV were also maximized at a threshold of 0.3 (95% and 73%, respectively) (Figure S-10). Combined, these data suggest a meaningful change threshold range of 0.8 to 1.7 for stool consistency. The distribution-based analyses for stool consistency are presented in Table S-5.

## Discussion

The PFCSD is a novel PRO measure designed to assess core symptoms of FC in pediatric patients aged 6 to 17 years. This psychometric evaluation supports the test-retest reliability, construct validity, and responsiveness of SBM frequency rate and stool consistency scores derived from the PFCSD, underscoring the use of these for constructing primary and secondary endpoints in pediatric FC clinical trials. Additionally, meaningful within-patient change thresholds defined for SBM frequency rate and stool consistency scores provide important guidance for interpreting changes on these scores at the individual level.

The heterogeneity of symptoms across patients with FC highlights the complexity of accurately capturing symptoms, which can be more challenging in the pediatric population due to various reading, comprehension, and attention levels. Indeed, inconsistent reliability and validity has been reported for PRO measures administered to children younger than 8 years [15]. To address this, 2 versions of the PFCSD were developed—a self-completed and a caregiver-administered version—each containing identical items that evaluated key symptoms in pediatric patients with FC. The PFCSD was found to be capable of consistently measuring SBM frequency rate and stool consistency (the key endpoints of the LIN-MD-64 trial) across repeated administrations in pediatric patients, regardless of mode of administration. Through construct validity analyses, the SBM frequency rate and stool consistency scores were found to measure the intended constructs and were distinct from abdominal symptom scores. Furthermore, the ability of the SBM frequency rate and stool consistency scores derived from the PFCSD to detect change over time was confirmed. Thus, these findings support the use of the PFCSD to obtain consistent and accurate self-reporting from pediatric patients with FC independent of age level or administration mode, which ultimately enables the assessment of treatment effects on key symptoms important to patients in this target population.

While statistical significance between groups may be used to determine treatment efficacy, this may not reflect changes in FC that are meaningful from the patient perspective. By establishing meaningful change thresholds for PRO scores, improvement that is meaningful at the individual level can be defined [16]. Since SBM frequency rate and stool consistency were the key endpoints in the LIN-MD-64 trial, meaningful within-patient change thresholds for these 2 PFCSD item scores were the focus of this psychometric evaluation. Based on triangulation across eCDFs and classification statistics, meaningful change thresholds were estimated to be a change from baseline of ≥ 2 SBMs per week and a change from baseline of 0.8 to 1.7 on the pediatric Bristol Stool Form Scale for stool consistency. Overall, this analysis provided interpretable scores for SBM frequency and stool consistency when assessing treatment benefit in clinical trials of pediatric patients with FC.

The ability to interpret certain data points was limited by the study inclusion criteria and small sample size. The study inclusion criteria for SBM frequency rate and stool consistency likely reduced the variability of these scores prior to randomization (Week − 2 to Week − 1), which could account for the reduced test-retest reliability at that timepoint. The small sample size for some of these psychometric analyses was largely driven by missing responses on the anchor variables, which may have been because those weekly items had to be accessed in a different location from the daily items within the PFCSD. As pediatric patients may have had difficulty accurately recalling changes in their health status over a 7-day period, a potential limitation of the weekly anchor items was the 7-day recall period. However, qualitative research in this target population supports the use of the 7-day recall period for both the PGIS and PGIC measures. Additionally, although caregivers were trained on the use and completion of the caregiver-administered version of the PFCSD, it is impossible to determine whether participants’ responses may have been influenced by the interviewer. However, the availability of the 2 administration modes enabled use of the PFCSD across all participants, regardless of their reading or comprehension level, and a strength of this study was the application of a fit-for-purpose measure developed for this context of use and patient population. Finally, no formal assessments against underlying assumptions in any of the statistical analyses (e.g., 2-way mixed effects model, analysis of variance) were conducted. As such, potential deviations from these assumptions could affect the statistical analyses.

## Conclusion

The PFCSD is a novel, fit-for-purpose PRO instrument developed to assess core symptoms experienced by patients with FC aged 6 to 17 years. This psychometric evaluation provides sufficient evidence for the reliability, validity, and responsiveness of the SBM frequency rate and stool consistency scores based on the PFCSD in a pediatric population aged 6 to 17 years with FC. Evidence generated supports future use of the PFCSD in defining endpoints and interpreting clinically meaningful change in SBM frequency rate and stool consistency scores to assess treatment benefit in pediatric FC trials.

## Supplementary Information

Below is the link to the electronic supplementary material.

Supplementary Material 1

## Data Availability

The data can be requested by any qualified researchers who engage in rigorous, independent, scientific research and will be provided following review and approval of a research proposal, Statistical Analysis Plan, and execution of a Data Sharing Agreement. Data requests can be submitted at any time after approval in the US and Europe and after acceptance of this manuscript for publication. The data will be accessible for 12 months, with possible extensions considered. For more information on the process or to submit a request, visit the provided link (https://vivli.org/ourmember/abbvie/​), then select “Home.”

## Abbreviations

BM: Bowel movement
CI: Confidence interval
CSBM: Complete spontaneous bowel movement
eCDF: Empirical cumulative distribution function
eDiary: Electronic Diary
FC: Functional constipation
FDA: US Food and Drug Administration
GRS: Guyatt’s responsiveness statistic
ICC: Intraclass correlation coefficient
NPV: Negative predictive value
PDF: Probability density function
PFCSD: Pediatric Functional Constipation Symptom Diary
PGIC: Patient Global Impression of Change
PGIS: Patient Global Impression of Severity
PPV: Positive predictive value
PRO: Patient-reported outcome
SBM: Spontaneous bowel movement
SD: Standard deviation
