# Correction: Psychometric evaluation of a pediatric functional constipation symptom diary using randomized phase 3 clinical trial data

**DOI:** 10.1186/s41687-026-01010-7

**Published:** 2026-03-02

**Authors:** Julia Vishnevetsky, Masakazu Ando, Yanqing Xu, Xiaolan Ye, Jessica L. Abel

**Affiliations:** 1 AbbVie, Inc., Florham Park, NJ USA; 2 Ironwood Pharmaceuticals, Boston, MA USA; 3 AbbVie, Inc., North Chicago, IL USA

**Correction: Journal of Patient-Reported Outcomes (2025) 9:134**

10.1186/s41687-025-00945-7

In this article the author name Daniel Williamson was incorrectly included in the author group and it is now removed from the author list. 

