# Supplementary material for: Psychometric evaluation of a pediatric functional constipation symptom diary using randomized phase 3 clinical trial data
Source: J Patient Rep Outcomes. 2025 Nov 20;9:134. doi: 10.1186/s41687-025-00945-7 (PMC12635004; doi:10.1186/s41687-025-00945-7)
Supplement: Supplementary file 1 — Supplementary Material 1 [file 41687_2025_945_MOESM1_ESM.docx]

Psychometric Evaluation of a Pediatric Functional Constipation Symptom Diary Using Randomized Phase 3 Clinical Trial Data

**Supplementary Material**

**Table S-1. Construct Validity Using Known-Groups Method With the Caregiver-Observed Severity Anchor**

| **PFCSD item** | **Time** | **Groups** | **N** | **Mean** | **SD** | **Effect size** | ***P* value** |
| --- | --- | --- | --- | --- | --- | --- | --- |
| SBM frequency rate | Week -1 | High severity | 56 | 1.27 | 1.21 | 0.04 | 0.111 |
|  |  | Low severity | 11 | 1.91 | 1.14 |  |  |
|  | Week 12 | High severity | 7 | 0.71 | 0.76 | 0.15 | 0.007 |
|  |  | Low severity | 41 | 3.59 | 2.65 |  |  |
| Stool consistency | Week -1 | High severity | 38 | 2.59 | 1.44 | 0.01 | 0.466 |
|  |  | Low severity | 10 | 2.23 | 1.06 |  |  |
|  | Week 12 | High severity | 4 | 3.13 | 2.66 | 0.03 | 0.259 |
|  |  | Low severity | 35 | 3.91 | 1.11 |  |  |
| CSBM frequency rate | Week -1 | High severity | 56 | 0.63 | 1.04 | 0.10 | 0.010 |
|  |  | Low severity | 11 | 1.55 | 1.13 |  |  |
|  | Week 12 | High severity | 7 | 0.43 | 0.79 | 0.15 | 0.007 |
|  |  | Low severity | 41 | 3.15 | 2.51 |  |  |
| Fecal incontinence | Week -1 | High severity | 56 | 0.10 | 0.16 | 0.00 | 0.773 |
|  |  | Low severity | 11 | 0.12 | 0.28 |  |  |
|  | Week 12 | High severity | 7 | 0.07 | 0.13 | 0.00 | 0.689 |
|  |  | Low severity | 41 | 0.05 | 0.15 |  |  |
| Straining | Week -1 | High severity | 39 | 2.66 | 1.17 | 0.21 | <0.001 |
|  |  | Low severity | 10 | 1.19 | 1.09 |  |  |
|  | Week 12 | High severity | 4 | 2.75 | 1.26 | 0.23 | 0.002 |
|  |  | Low severity | 36 | 1.00 | 0.95 |  |  |
| Abdominal pain | Week -1 | High severity | 56 | 1.28 | 1.23 | 0.09 | 0.013 |
|  |  | Low severity | 11 | 0.31 | 0.35 |  |  |
|  | Week 12 | High severity | 7 | 1.27 | 1.57 | 0.14 | 0.010 |
|  |  | Low severity | 41 | 0.35 | 0.66 |  |  |
| Abdominal bloating | Week -1 | High severity | 56 | 1.32 | 1.25 | 0.07 | 0.029 |
|  |  | Low severity | 11 | 0.45 | 0.67 |  |  |
|  | Week 12 | High severity | 7 | 1.49 | 1.25 | 0.26 | <0.001 |
|  |  | Low severity | 41 | 0.26 | 0.65 |  |  |

CSBM = complete SBM; PFCSD = Pediatric Functional Constipation Symptom Diary; SBM = spontaneous bowel movement; SD = standard deviation

Notes: Severity groups were defined by the caregiver-observed global severity anchor item (“constipation severity”) for Week -1 and Week 12. High severity is defined as a response of “severe” or “very severe.” Low severity is defined as a response of “none” or “mild.”

**Table S-2. Responsiveness Statistics for Change in PFCSD Scores From Week 11 to Week 12 Using the PGIC Anchors**

| **PFCSD item** | **Groups** | **N** | **Mean** | **SD** | **GRS improved vs. stable** | **GRS improved vs. worsened** |
| --- | --- | --- | --- | --- | --- | --- |
| SBM frequency rate | Improved | 37 | -0.86 | 2.43 | -0.43 | -0.45 |
|  | Stable | 35 | 0.20 | 2.47 |  |  |
|  | Worsened | 7 | 0.00 | 1.91 |  |  |
| Stool consistency | Improved | 33 | -0.02 | 0.94 | 0.19 | 0.41 |
|  | Stable | 24 | -0.25 | 1.24 |  |  |
|  | Worsened | 3 | -0.28 | 0.63 |  |  |
| CSBM frequency rate | Improved | 37 | -0.59 | 2.41 | -0.39 | -1.80 |
|  | Stable | 35 | 0.34 | 2.41 |  |  |
|  | Worsened | 7 | 0.29 | 0.49 |  |  |
| Fecal incontinence | Improved | 37 | -0.02 | 0.10 | -0.19 | -0.69 |
|  | Stable | 35 | 0.00 | 0.12 |  |  |
|  | Worsened | 7 | 0.11 | 0.18 |  |  |
| Straining | Improved | 33 | -0.07 | 0.68 | 0.05 | 0.46 |
|  | Stable | 24 | -0.12 | 1.01 |  |  |
|  | Worsened | 3 | -0.33 | 0.58 |  |  |
| Abdominal pain | Improved | 32 | 0.01 | 0.29 | 0.02 | 0.15 |
|  | Stable | 39 | 0.00 | 0.48 |  |  |
|  | Worsened | 8 | -0.06 | 0.49 |  |  |
| Abdominal bloating | Improved | 32 | 0.04 | 0.21 | 0.10 | 0.12 |
|  | Stable | 39 | 0.01 | 0.33 |  |  |
|  | Worsened | 8 | -0.02 | 0.48 |  |  |

CSBM = complete SBM; GRS = Guyatt’s responsiveness statistic; PFCSD = Pediatric Functional Constipation Symptom Diary; PGIC = Patient Global Impression of Change; SBM = spontaneous bowel movement; SD = standard deviation

Notes: PGIC items correspond to the PFCSD items as follows: “tummy problems” for abdominal pain and abdominal bloating PFCSD scores, and “pooping problems” for the remaining PFCSD scores. Change is computed as the later time point (improved) minus the earlier time point (stable or worsened).

**Table S-3. Responsiveness Statistics for Change in PFCSD Scores From Week -1 to Week 12 Using the Caregiver-Observed Severity Anchor**

| **PFCSD item** | **Groups** | **N** | **Mean** | **SD** | **GRS improved vs. stable** | **GRS improved vs. worsened** |
| --- | --- | --- | --- | --- | --- | --- |
| SBM frequency rate | Improved | 39 | 1.92 | 2.49 | 0.40 | 0.27 |
|  | Stable | 16 | 0.75 | 2.96 |  |  |
|  | Worsened | 3 | 0.67 | 4.73 |  |  |
| Stool consistency | Improved | 21 | 1.58 | 1.22 | 2.64 | 7.51 |
|  | Stable | 7 | -0.15 | 0.65 |  |  |
|  | Worsened | 2 | -0.32 | 0.25 |  |  |
| CSBM frequency rate | Improved | 39 | 2.33 | 2.56 | 0.78 | 0.31 |
|  | Stable | 16 | 0.69 | 2.12 |  |  |
|  | Worsened | 3 | 1.00 | 4.36 |  |  |
| Fecal incontinence | Improved | 39 | -0.01 | 0.16 | 0.27 | -0.69 |
|  | Stable | 16 | -0.08 | 0.25 |  |  |
|  | Worsened | 3 | 0.05 | 0.08 |  |  |
| Straining | Improved | 21 | -1.50 | 1.59 | -1.38 | -2.83 |
|  | Stable | 8 | -0.07 | 1.04 |  |  |
|  | Worsened | 2 | 0.50 | 0.71 |  |  |
| Abdominal pain | Improved | 39 | -0.51 | 0.98 | -1.02 | -3.61 |
|  | Stable | 16 | -0.09 | 0.42 |  |  |
|  | Worsened | 3 | -0.12 | 0.11 |  |  |
| Abdominal bloating | Improved | 39 | -0.58 | 0.86 | -0.84 | -4.17 |
|  | Stable | 16 | -0.14 | 0.53 |  |  |
|  | Worsened | 3 | 0.17 | 0.18 |  |  |

CSBM = complete SBM; GRS = Guyatt’s responsiveness statistic; PFCSD = Pediatric Functional Constipation Symptom Diary; SBM = spontaneous bowel movement; SD = standard deviation.

Notes: The caregiver-observed severity anchor is based on the child's constipation severity for all PFCSD scores and is available only for participants 6-11 years of age. Change is computed as the later time point (improved) minus the earlier time point (stable or worsened). Improved = one category improvement or greater. Stable = no change. Worsened = one category worsened or greater

**Table S-4. Responsiveness Statistics for Change in PFCSD Scores From Week 11 to Week 12 Using the Caregiver-Observed Change Anchor**

| **PFCSD item** | **Groups** | **N** | **Mean** | **SD** | **GRS improved vs. stable** |
| --- | --- | --- | --- | --- | --- |
| SBM frequency rate | Improved | 19 | -0.79 | 3.54 | -0.17 |
|  | Stable | 16 | -0.50 | 1.75 |  |
| Stool consistency | Improved | 19 | 0.03 | 0.55 | 0.08 |
|  | Stable | 9 | -0.07 | 1.21 |  |
| CSBM frequency rate | Improved | 19 | -0.68 | 3.54 | -0.07 |
|  | Stable | 16 | -0.56 | 1.71 |  |
| Fecal incontinence | Improved | 19 | -0.03 | 0.10 | -0.45 |
|  | Stable | 16 | 0.01 | 0.10 |  |
| Straining | Improved | 19 | 0.01 | 0.45 | -0.48 |
|  | Stable | 9 | 0.19 | 0.39 |  |
| Abdominal pain | Improved | 19 | 0.02 | 0.29 | 0.21 |
|  | Stable | 16 | -0.07 | 0.43 |  |
| Abdominal bloating | Improved | 19 | -0.01 | 0.23 | -0.48 |
|  | Stable | 16 | 0.06 | 0.14 |  |

CSBM = complete SBM; GRS = Guyatt’s responsiveness statistic; PFCSD = Pediatric Functional Constipation Symptom Diary; SBM = spontaneous bowel movement; SD = standard deviation.

Notes: The caregiver-observed change anchor is based on the child's constipation symptoms “today” compared with the previous 7 days for all PFCSD scores and is available only for participants 6-11 years of age. The Worsened group for each PFCSD item had a sample size of 1. Thus, the mean, SD, and GRS of Improved vs. Worsened could not be calculated.

**Table S-5. Distribution-Based Estimates for SBM Frequency Rate and Stool Consistency PFCSD Scores Using Standard Deviations and Test-Retest Reliability**

| **PFCSD Item** | **½ SD**  **Weeks -1 through -2** | | | **SEM**  **Week -2 to Week -1** | | | **SEM**  **Week 11 to Week 12** | | | |
| --- | --- | --- | --- | --- | --- | --- | --- | --- | --- | --- |
|  | **N** | **½ SD** | **N** | | **ICC** | **SEM** | | **N** | **ICC** | **SEM** |
| SBM frequency rate | 328 | 0.42 | 114 | | 0.05 | 0.83 | | 57 | 0.91 | 0.25 |
| Stool consistency | 276 | 0.47 | 58 | | 0.35 | 0.75 | | 45 | 0.56 | 0.62 |

ICC = intraclass correlation coefficient; PFCSD = Pediatric Functional Constipation Symptom Diary; SBM = spontaneous bowel movement; SD = standard deviation; SEM = standard error of measurement.

Note: The reliability used in the SEM computation is based on the test-retest reliability ICC estimates from Table 3.

**Figure S-1. PDF of PFCSD SBM Frequency Rate Change From Week -1 to Week 12 and PGIS Pooping Problems Item Change From Week -1 to Week 12**


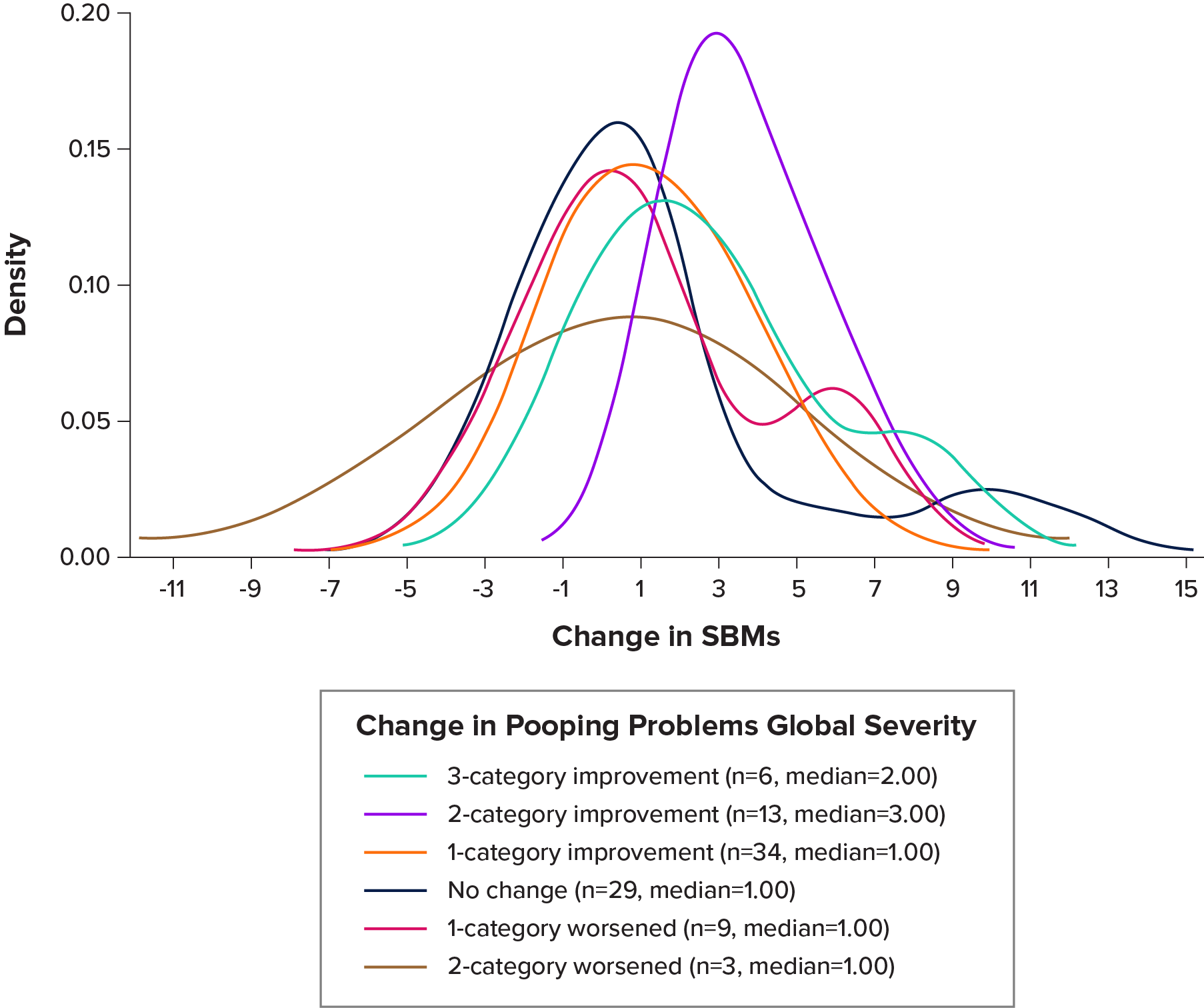


PGIS = Patient Global Impression of Severity

Notes: Change was computed as the later time point minus the earlier time point. Positive change indicates improvement. The pooping problems PGIS item was self-completed and available for all participants.

**Figure S-2. Classification Statistics for PFCSD SBM Frequency Rate Change From Week -1 to Week 12** **Using PGIS Pooping Problems Item Change From Week -1 to Week 12**

**
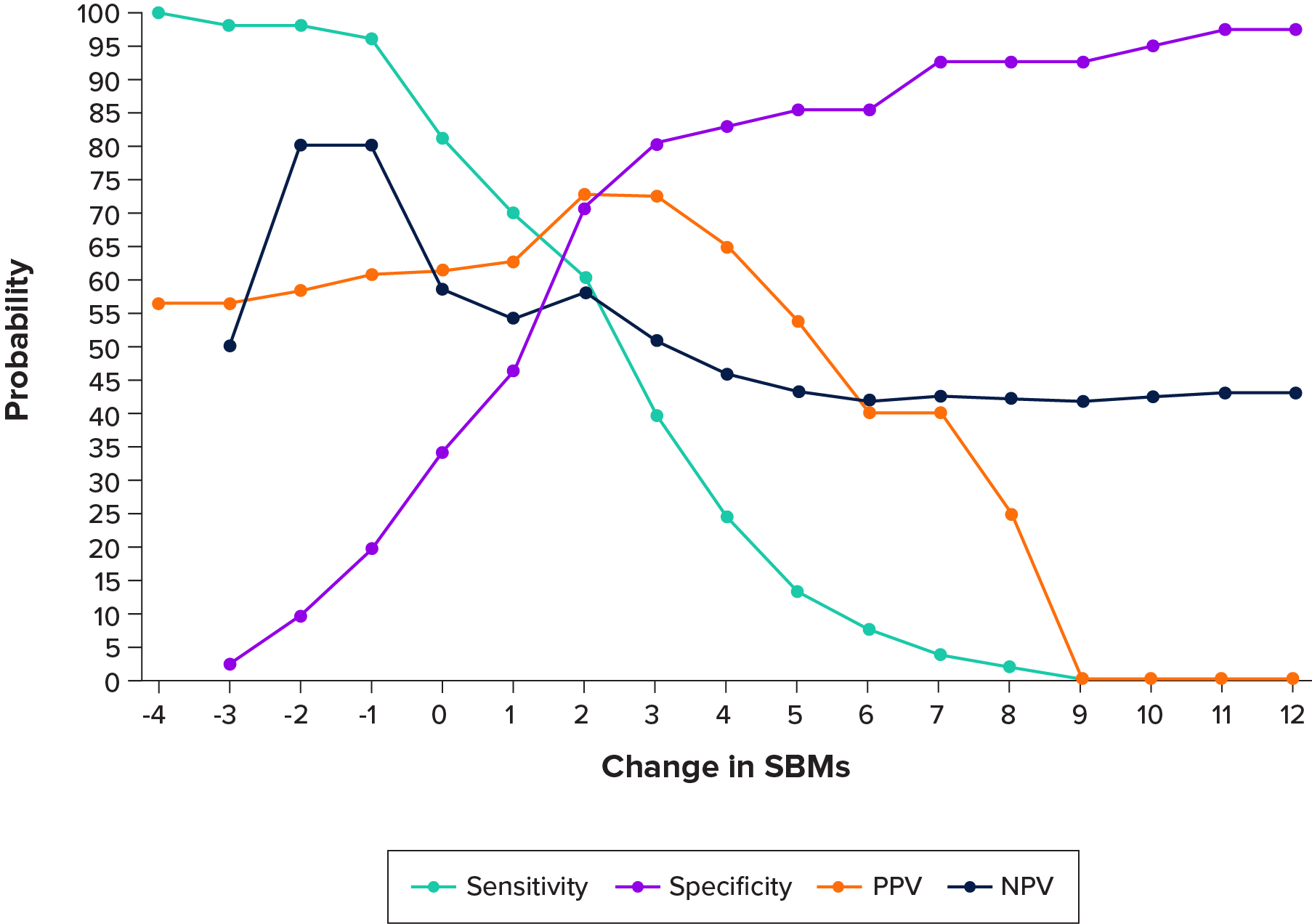
**

PGIS = Patient Global Impression of Severity

Notes: Change was computed as the later time point minus the earlier time point. Positive change indicates improvement. The pooping problems PGIS was self-completed and available for all participants. Meaningful improvement was defined as a 1-category improvement or more. n = 94.

**Figure S-3. eCDF of PFCSD SBM Frequency Rate Change From Week -1 to Week 12 and Constipation Global Severity Change From Week -1 to Week 12**


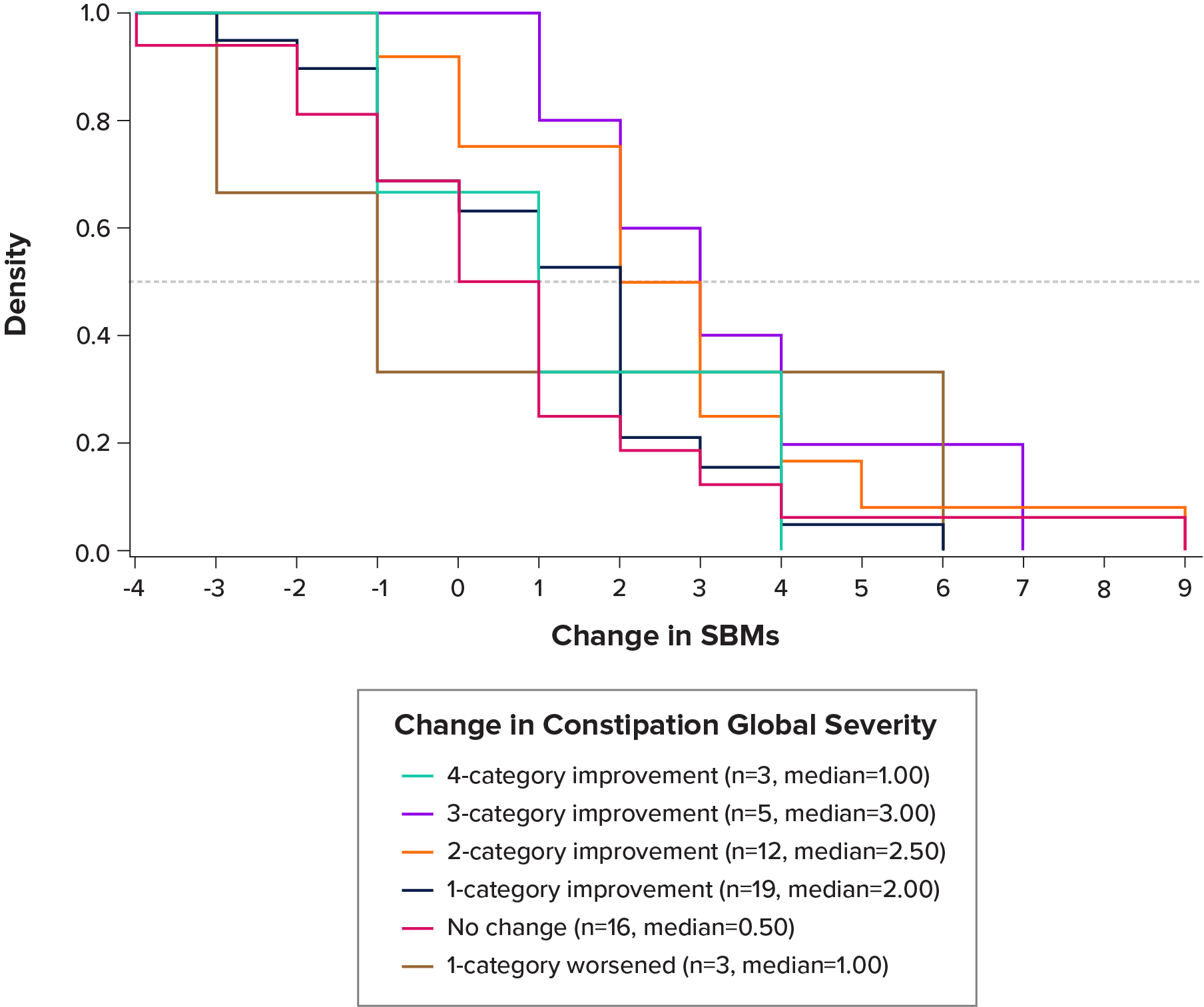


Notes: Change was computed as the later time point minus the earlier time point. Positive change indicates improvement. Constipation severity was caregiver observed and was available only for participants 6-11 years of age.

**Figure S-4. PDF of PFCSD SBM Frequency Rate Change From Week -1 to Week 12 and Constipation Global Severity Change From Week -1 to Week 12**


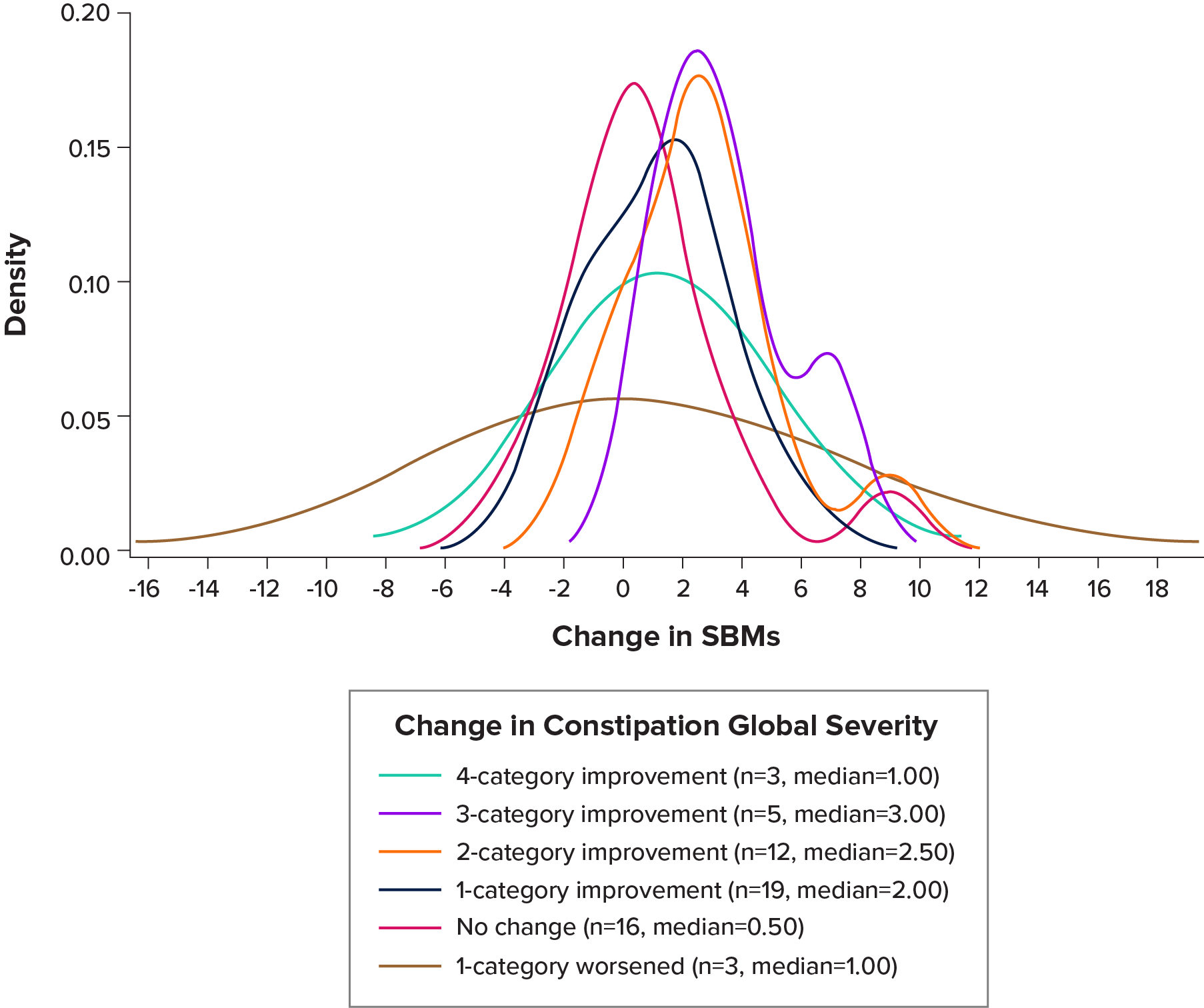


Notes: Change was computed as the later time point minus the earlier time point. Positive change indicates improvement. Constipation severity was caregiver observed and was available only for participants 6-11 years of age.

**Figure S-5. Classification Statistics for PFCSD SBM Frequency Rate Change From Week -1 to Week 12 Using Constipation Global Severity Change From Week -1 to Week 12**


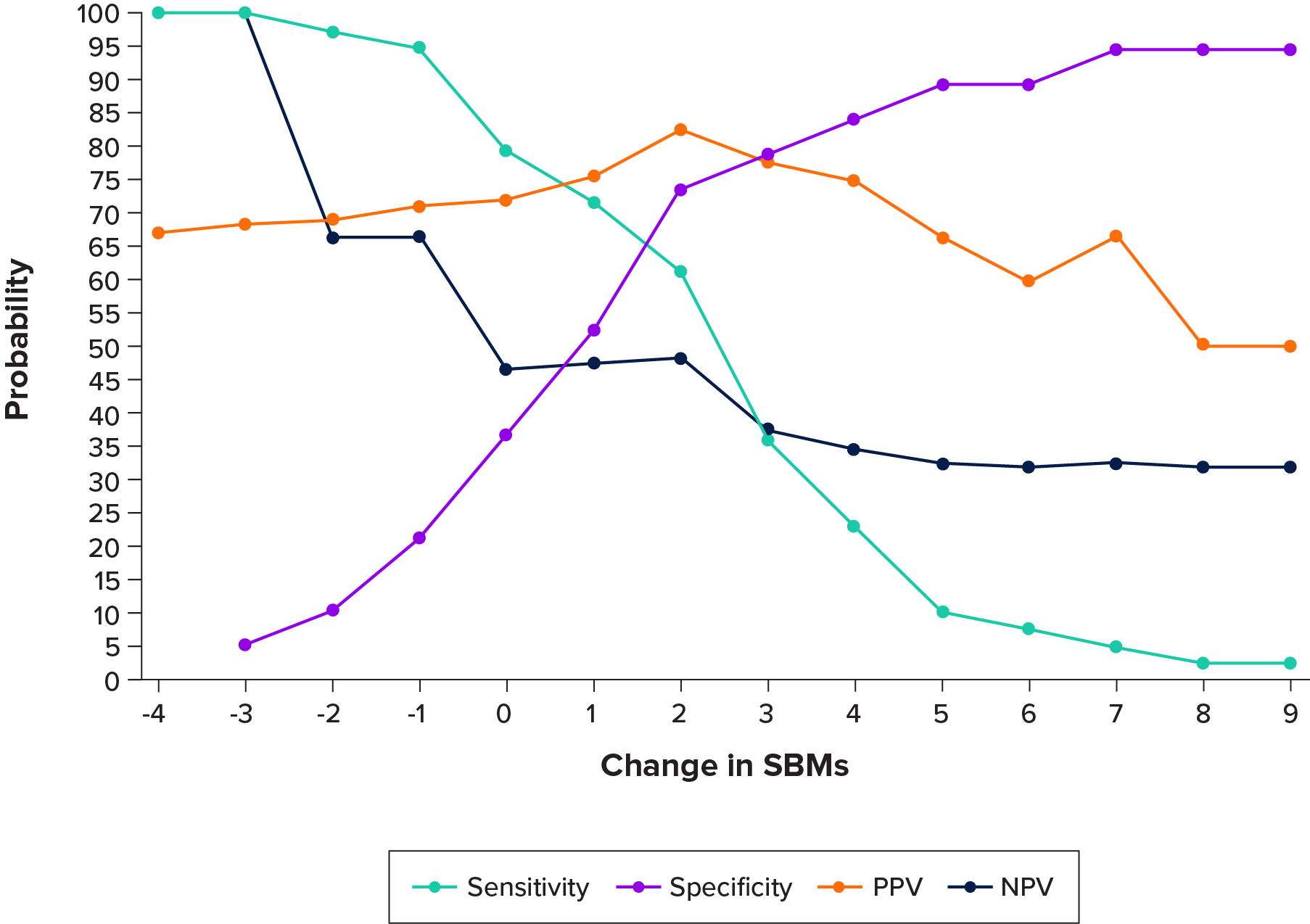


Notes: Change was computed as the later time point minus the earlier time point. Positive change indicates improvement. Constipation severity was caregiver observed and was available only for participants 6-11 years of age. Meaningful improvement was defined as a 1-category improvement or more. n = 58.

**Figure S-6. PDF of PFCSD Stool Consistency Change From Week -1 to Week 12 and PGIS Pooping Problems Item Change From Week -1 to Week 12**


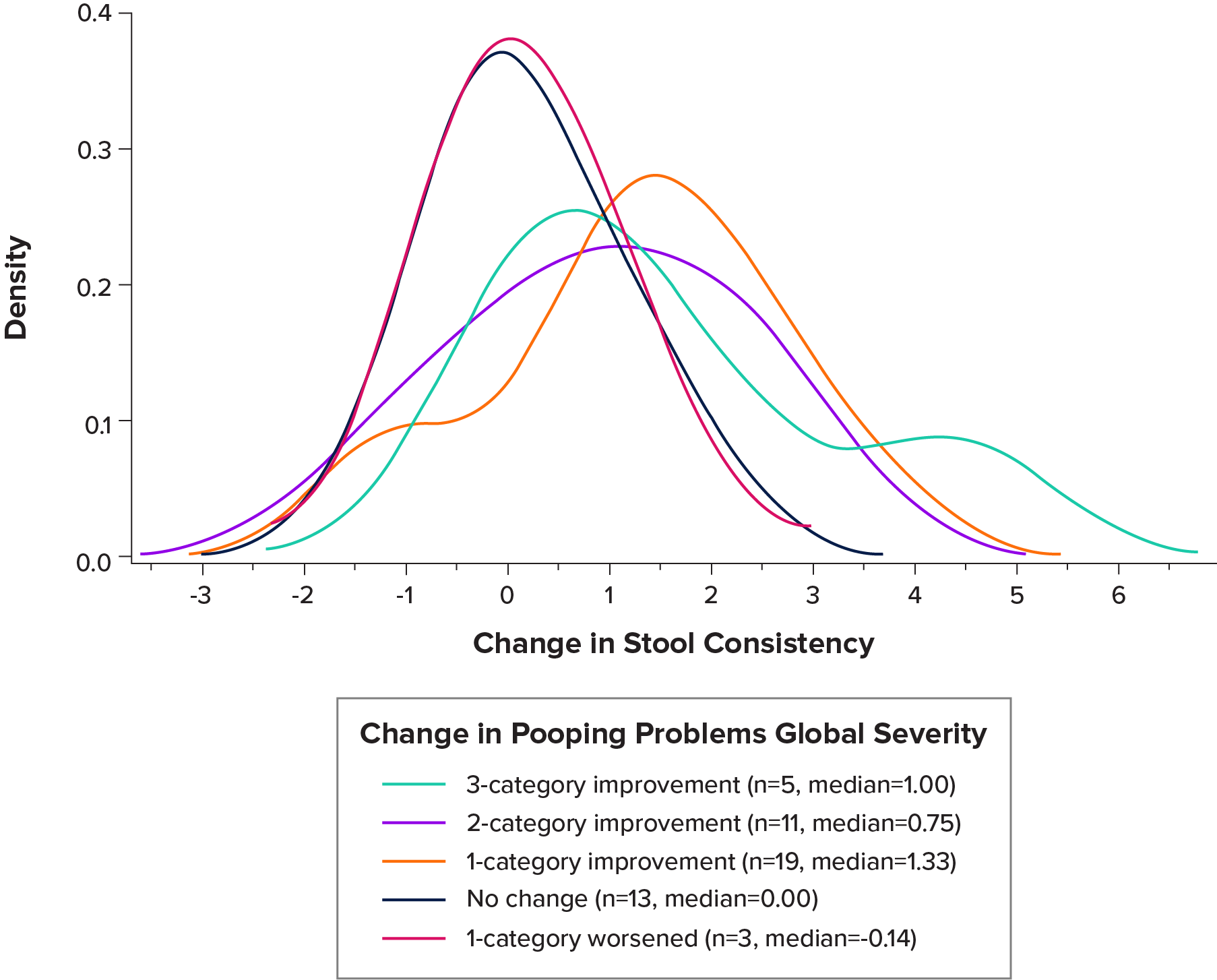


PGIS = Patient Global Impression of Severity

Notes: Change was computed as the later time point minus the earlier time point. Positive change indicates improvement. The pooping problems PGIS item was self-completed and available for all participants.

**Figure S-7. Classification Statistics for PFCSD Stool Consistency Change From Week -1 to Week 12 and PGIS Pooping Problems Item Change From Week -1 to Week 12**


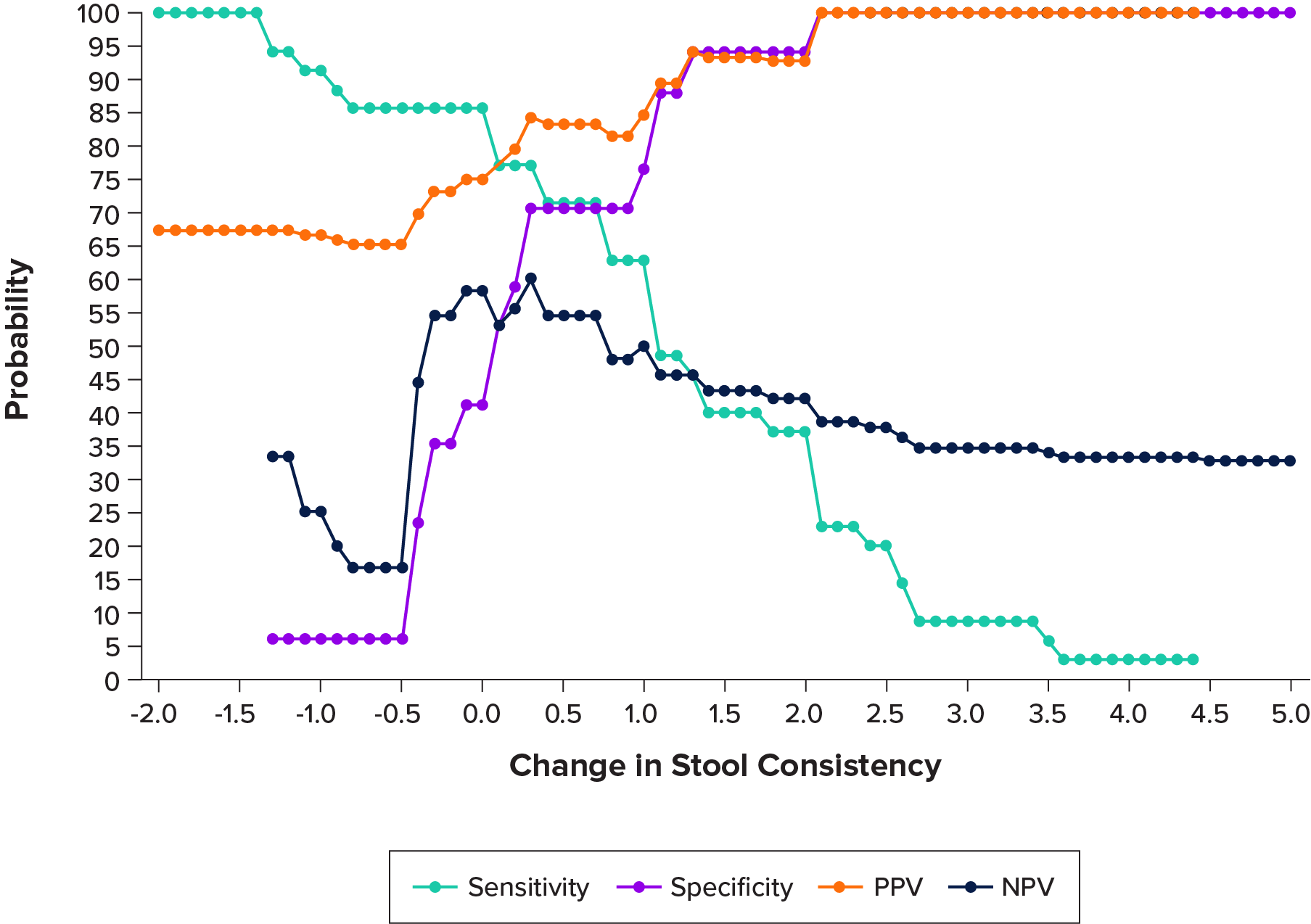


PGIS = Patient Global Impression of Severity

Notes: Change was computed as the later time point minus the earlier time point. Positive change indicates improvement. The pooping problems PGIS item was self-completed and available for all participants. Meaningful improvement was defined as a 1-category improvement or more. n = 52.

**Figure S-8. eCDF of PFCSD Stool Consistency Change From Week -1 to Week 12 and Constipation Global Severity Change From Week -1 to Week 12**


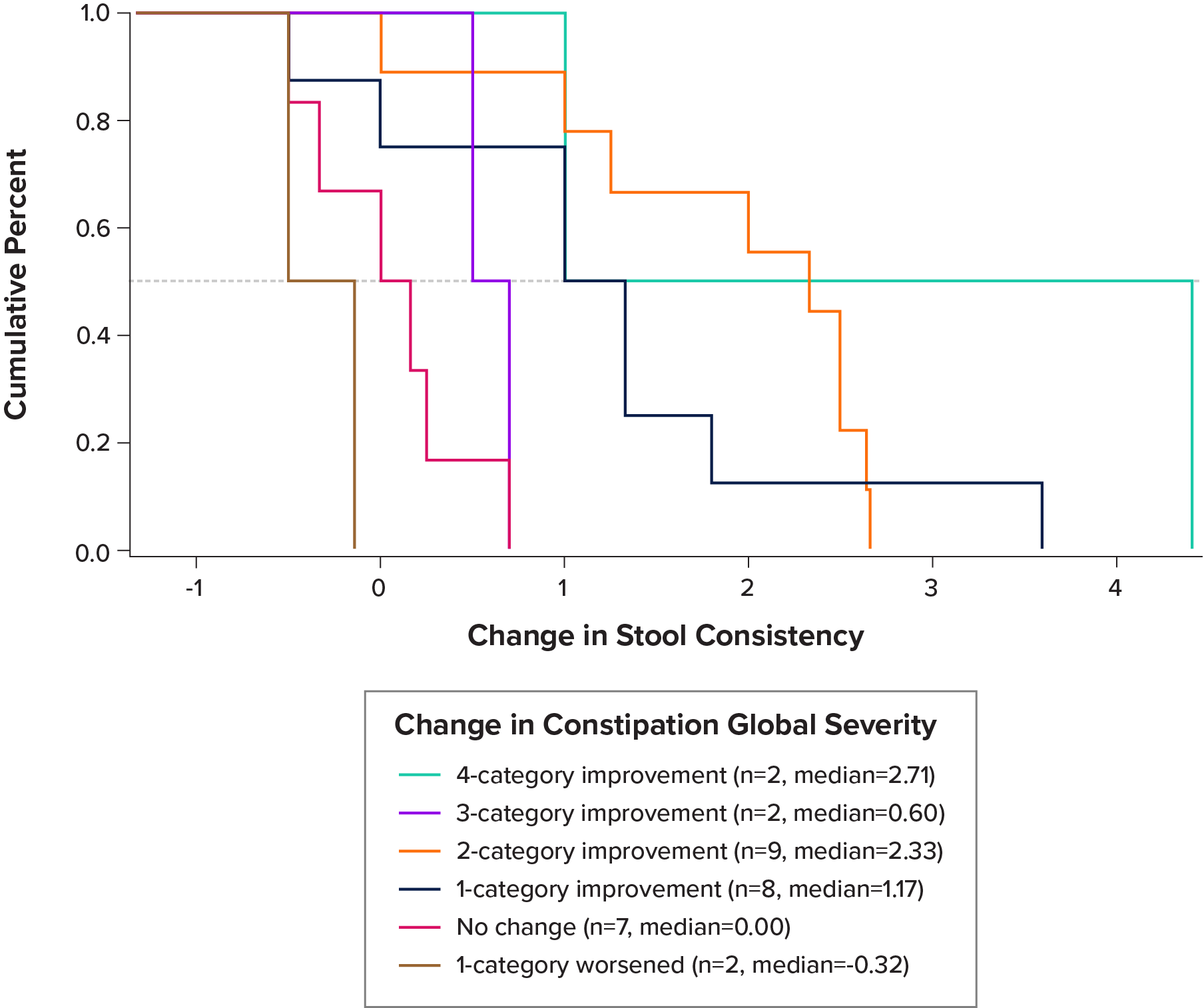


Notes: Change was computed as the later time point minus the earlier time point. Positive change indicates improvement. Constipation severity was caregiver observed and was available only for participants 6-11 years of age.

**Figure S-9. PDF of PFCSD Stool Consistency Change From Week -1 to Week 12 and Constipation Global Severity Change From Week -1 to Week 12**


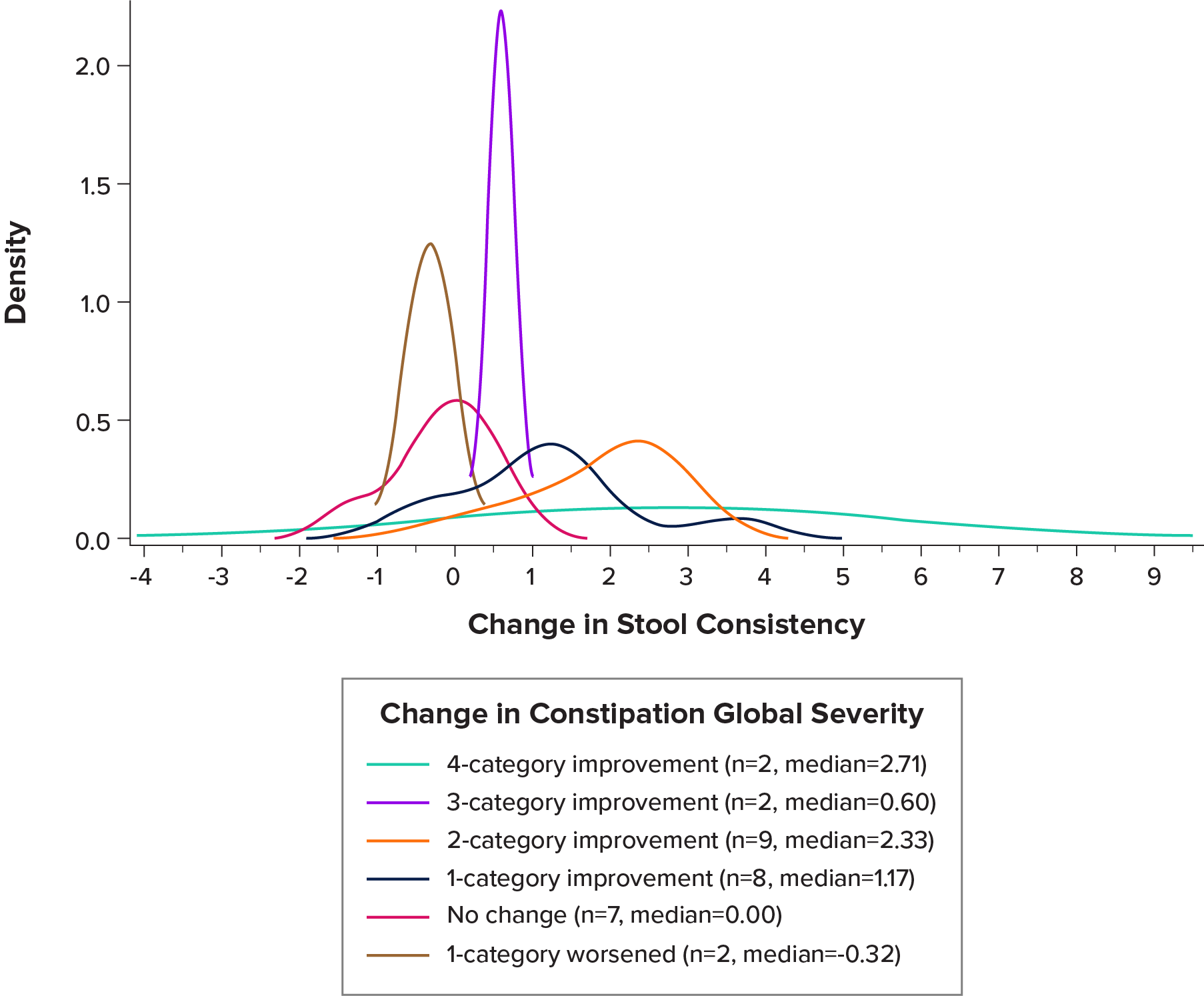


Notes: Change was computed as the later time point minus the earlier time point. Positive change indicates improvement. Constipation severity was caregiver-observed and was available only for participants 6-11 years of age.

**Figure S-10. Classification Statistics for PFCSD Stool Consistency Change From Week -1 to Week 12 Using Constipation Global Severity Change From Week -1 to Week 12**


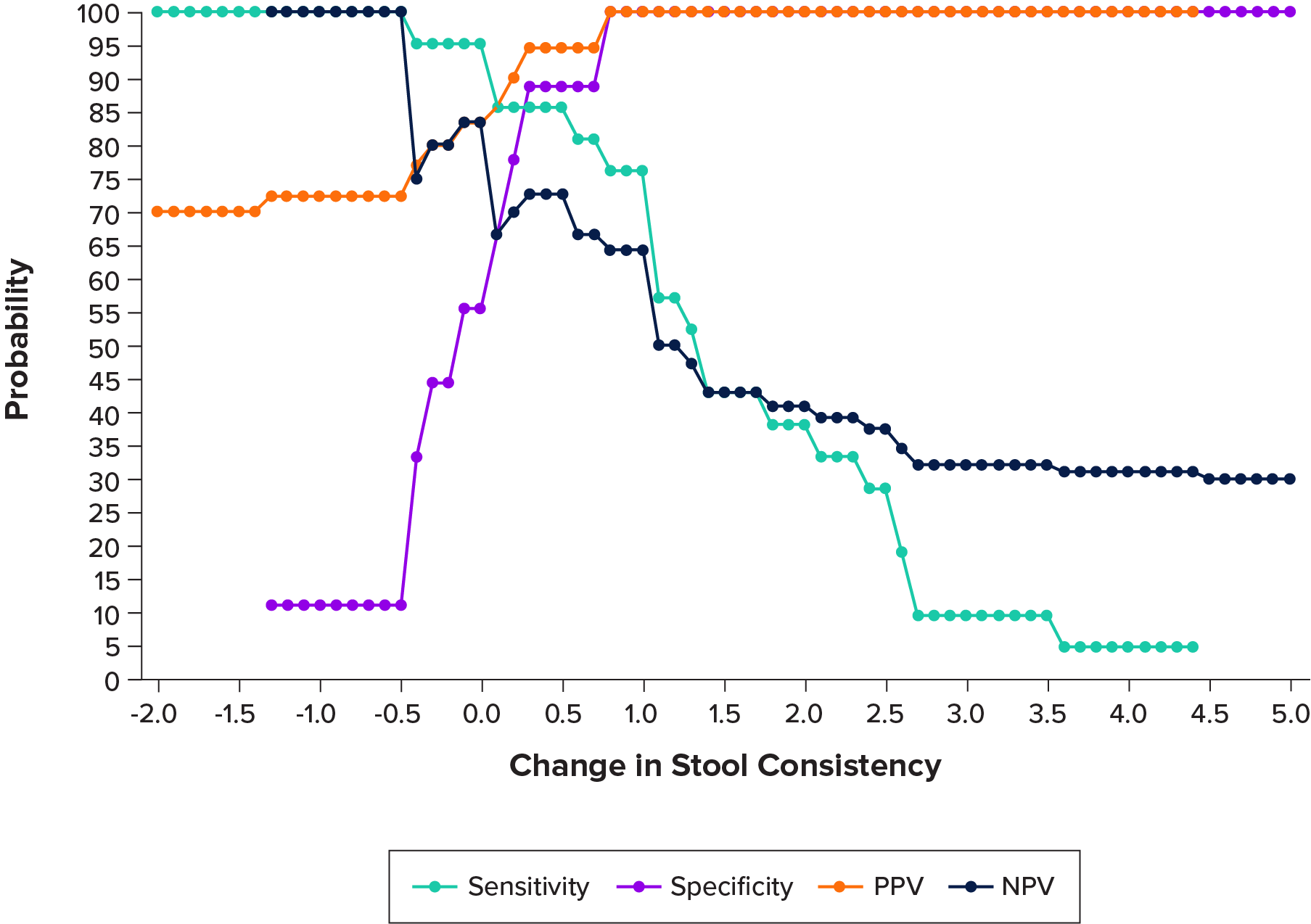


Notes: Change was computed as the later time point minus the earlier time point. Positive change indicates improvement. Constipation severity was caregiver-observed and was available only for participants 6-11 years of age. Meaningful improvement was defined as a 1-category improvement or more. n= 30.
